# Common computational tools for analyzing CRISPR screens

**DOI:** 10.1042/ETLS20210222

**Published:** 2021-12-09

**Authors:** Medina Colic, Traver Hart

**Affiliations:** 1 Department of Bioinformatics and Computational Biology, The University of Texas MD Anderson Cancer Center, Houston, TX 77030, U.S.A.; 2 UTHealth Graduate School of Biomedical Sciences, The University of Texas MD Anderson Cancer Center, Houston, TX, U.S.A.; 3 Department of Cancer Biology, The University of Texas MD Anderson Cancer Center, Houston, TX 77030, U.S.A.

**Keywords:** bioinformatcis, CRISPR, drug targeting

## Abstract

CRISPR–Cas technology offers a versatile toolbox for genome editing, with applications in various cancer-related fields such as functional genomics, immunotherapy, synthetic lethality and drug resistance, metastasis, genome regulation, chromatic accessibility and RNA-targeting. The variety of screening platforms and questions in which they are used have caused the development of a wide array of analytical methods for CRISPR analysis. In this review, we focus on the algorithms and frameworks used in the computational analysis of pooled CRISPR knockout (KO) screens and highlight some of the most significant target discoveries made using these methods. Lastly, we offer perspectives on the design and analysis of state-of-art multiplex screening for genetic interactions.

## Introduction

**Figure 1.**
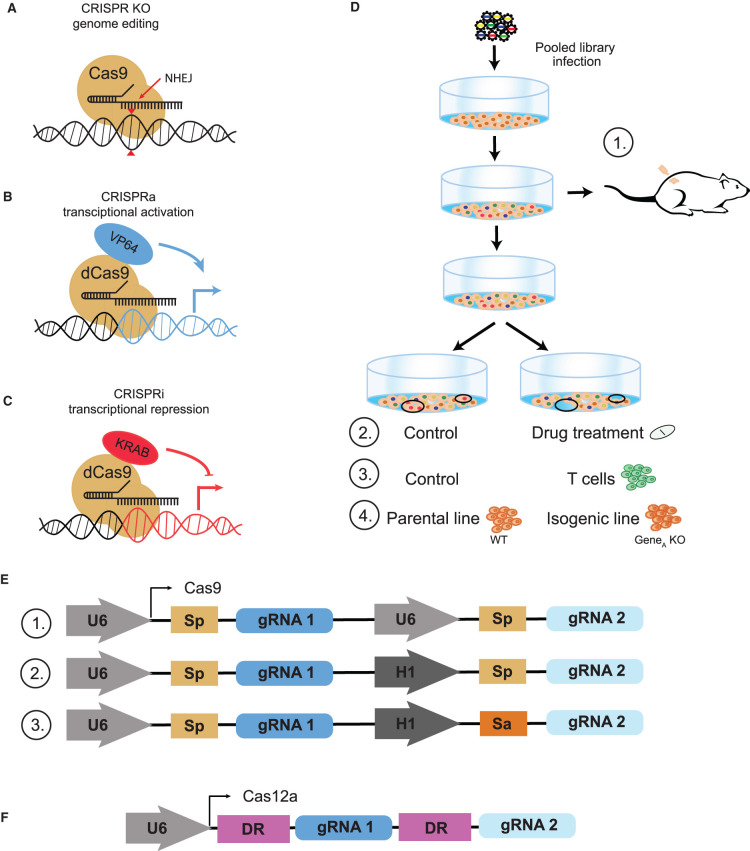
CRISPR Toolbox. (**A**) CRISPR knockout. (**B**) CRISPR activation. (**C**) CRISPR interferance, (**D**) Pooled screens: (**1**) *In vivo*, (**2**) Chemogenetic, (**3**) Immuno-oncology, and (**4**) Isogenic screens. (**E**) Cas9 multiplex platforms: (**1**) Single Cas9 (e.g. S. pyogenes) system using two copies of the U6 promoter. (**2**) Single Cas9 system uses two different promoters. (**3**) A two Cas9, two different promoters system. (**F**) EnCas12a multiplex platform.

The last decade highlights the new genome editing technology repurposed from a bacterial adaptive immune system [1] and its adaptation to mammalian genome engineering [2,3]. The CRISPR–Cas system utilizes the Cas nuclease, which is guided to the target sequence by a short guide RNA molecule (gRNA), where it introduces a double-strand break at the desired locus (Figure 1A). Activation of error-prone repair by nonhomologous end-joining pathways (NHEJ) results in a frameshift mutation creating a gene knockout (KO). When DNA damage is too great a burden on the model systems, alternative, engineered Cas approaches are available. Nuclease-inactivaed ‘dead' Cas9 (dCas9) can be fused with transcriptional activation or repression domains and targeted to gene promoters to activate (CRISPRa) or repress/inhibit (CRISPRi) gene transcription (Figure 1B,C). dCas9 systems have been reviewed in a greater detail by Kazi and Biswas [4].

Gene KO is the most widely used tool in the CRISPR toolkit. CRISPR KO screens answer how essential, or how necessary a gene is for a cellular fitness, with genes showing moderate to severe fitness defects often called ‘fitness genes' or ‘essential genes'. Exceptional examples of genome-wide CRISPR KO screens are two large pan-cancer CRISPR–Cas9 studies performed by the Broad Institute and the Wellcome Sanger Institutes [5,6], in which over a thousand cancer cell lines were screened with genome-scale KO screens. In addition to individual efforts, these two institutes work collaboratively [7–9] with an aim of creating a comprehensive map of all the intracellular genetic dependencies and vulnerabilities of cancer, known as the Cancer Dependency Map (DepMap) project [10,11]. Such efforts hold a premise of providing a comprehensive representation of cancer heterogeneity and an avenue for developing new therapies.

The emergence of CRISPR-mediated genetic screens and continued improvement in CRISPR reagent design [12–14] has enable investigation of genome-wide and custom libraries gene-drug interaction in human cells [15–27]. These studies illustrated the power of chemogenetic screens (CRISPR + drug perturbation) (Figure 1D) in identifying new genetic vulnerabilities to PARP, ATR, BRAF, NAMPT inhibitors, and temozolomide, and shed a light on using such experimental set-ups for a discovery of novel therapeutic targets. A more comprehensive overview of chemogenetic screens in human cancer cell lines can be found in a similar review [28].

In comparison with cell culture, *in vivo* systems are preferred for translational cancer research (e.g. evaluating tumor progression and therapeutic response), as they provide a more clinically relevant environment for tumor modeling. CRISPR editing in *in vivo* model in conducted by creating the mutant cell population of interest in a dish and then implanting those into a mouse, often subcutaneously or intravenously (Figure 1D). In the last few years, CRISPR technology has been used in living model organisms for studying various cancers and cancer specific processes [29–35], though the complexity of the approach limits these screens to targeted gene panels.

CRISPR screens are being used in immune-oncology studies as well, with the most common approach being to proliferate CRISPR-mutagenized cells in the presence or absence of T cells (or other immune system components) (Figure 1D). In the recent years, pooled CRISPR screens in tumor/immune co-culture systems have provided insights into tumor mechanisms that cause resistance to immunotherapies [36,37], genes involved in the immune synergistic interactions [36], and identification of novel targets for immune-oncology [38]. The studies described in the review focusing on interrogating immune cells and cancer with CRISPR–Cas9 [39] are the proof that the CRISPR screens are a powerful tool for investigating tumor–immune co-culture systems.

Though these approaches offer an enormous advantage over the prior state of the art, widespread genetic buffering imposes clear constraints on the ability of monogenic KO systems to provide saturating screens. These constraints have driven the development of multiplex targeting platforms via delivery of multiple sgRNAs per cell. This can be facilitated by using two Cas9 nucleases derived from different bacterial species, e.g. *S. pyogenes* and *S. aureus*, with species-specific gRNA expressed from different promoters (e.g. hU6 and mU6) (Figure 1E). Other systems use a single SpCas9 with two gRNA expressed from different promoters (Figure 1E). The research produced by these combined multiplex targeting systems has shown the potential to identify context-specific genetic interactions, candidate combinatorial drug treatments and potential drug targets [40–47], but library design and construction is highly complex.

Analysis of first-generation CRISPR screens was carried out with the help of methods primarily developed for the analysis of RNAi [48,49] and RNA-seq [50–53] data. However, it was noticeable that there was a need for computational methods which are considerate of sgRNA library size, design, and relevant controls, all of which are specific to CRISPR data. To address that need some groups have developed methods specific to the context of their studies, whereas other groups have developed more generic software and frameworks that are widely accepted and used by scientific community. In this review, we will provide an overview for the computational methods mainly for pooled CRISPR KO screens. Undoubtedly additional tools will be required as the experimental technology continues its rapid advances.

It is noticeable that there are common features across these methods such as the input data, which is typically a matrix populated with raw read counts, where rows are individual sgRNAs used in the library and headers are the samples and/or replicates in which the screens are carries out. An essential step in these analyses is the normalization of the raw read counts to prevent the comparisons of extreme values which could lead to the increase in false positives in the downstream analysis. The most commonly implemented normalization approaches in the methods mentioned in this review are based on mean, median and total read counts normalization factors. Another component that is accounted for in some of the methods is the number of replicates which directly relates to the modeling the distribution of sgRNAs, while some methods use the direct estimates of distribution parameters others use resampling approaches to smooth these estimates. Lastly, the hits in these screens are identified mainly by comparing the abundances of sgRNAs at the start versus the end of the experiment, or untreated versus treated samples, and where the corresponding analytical methods differ mostly is which modeling approach they use to quantify this difference in sgRNA abundance. An overview of computational methods widely used for the analysis of CRISPR-based pooled screens is presented in this section and a brief description for several others can be found in Table 1. The overview for each method includes the purpose of its design, brief explanation of its computational and mathematical design, as well as the most significant targets discovered or reconfirmed by it.

**Table 1:** Non web-based algorithms/methods for analysis of pooled CRISPR screens

Algorithm name	Description	Language
MAGeCK [54–56]	Negative binomial model — based analysis of genome-wide CRISPR–Cas9 KO screens for prioritizing sgRNAs, genes and pathways.	Python, R
HiTSelect [57]	Uses Poisson distribution to evaluate sgRNAs and stochastic multiobjective ranking method to generate gene-level statistics.	Matlab
ScreenBEAM [58]	Bayesian hierarchical (multilevel) model to directly assess gene-level activity from all relevant measurements.	R
STARS [12]	Gene-ranking algorithm for genetic perturbation screens — gene scores are computed using the probability mass function of a binomial distribution.	Python
BAGEL [59,60]	Bayesian analysis for identifying essential genes from pooled screens, based on core essential and nonessential gene sets.	Python
CaRpools [61]	A pipeline for end-to-end analysis of pooled CRISPR/Cas9 screening data. Including in-depth analysis of screening quality and sgRNA phenotypes.	R
CasTLE [62]	Maximum likelihood estimator and empirical Bayesian framework to account for multiple sources of variability, including reagent efficacy and off-target effects for the analysis of large-scale genomic perturbation screens.	Python
CERES [5]	A method to estimate gene dependency from essentiality screens while computationally correcting the copy number effect, therefore enabling unbiased interpretation of gene dependency at all levels of copy number.	R
ENCoRE [63]	Workflow for NGS to CRISPR gene results.	Java
PBNPA [64]	Permutation-based non-parametric analysis, which computes *P*-values at the gene level by permuting sgRNA labels, therefore avoids restrictive distributional assumptions.	R
CRISPhieRmix [65]	Broad-tailed null distribution is fit using negative control sgRNAs. Then, a mixture distribution is fit on all sgRNAs, ignoring gene identities. Lastly, using the mixture distribution the false discovery rate for each gene is calculated.	R
CB2 [66]	Beta-binomial model with a modified Student's *t*-test to measure differences in sgRNA levels, followed by Fisher's combined probability test to estimate the gene-level significance.	R
JACKS [67]	Bayesian method that jointly analyzes screens performed with the same library and assigns a gene *P*-value based on empirically derived null distribution based on essentiality scores in a known set of negative control genes.	Python
DrugZ [68]	Identifies synergistic and suppressor drug-gene interactions from CRISPR-based chemogenetic screens.	Python
Gscreend [69]	Mixture of a parametric null distribution is used to calculate *P*-value for every sgRNA, and robust rank aggregation (RRA) algorithm is used to aggregate and score the data on gene-level.	R
CRISPRcleanR [70]	Unsupervised copy number correction of gene-independent responses in genome wide CRISPR KO screens based on circular binary segmentation algorithm.	Python, R
CRISPy [71]	Supervised copy number correction of gene-independent effects, which uses Gaussian processes regression to model non-linear effects between the segment copy number ratio and CRISPR fold changes.	R

### Analysis of individual screens

#### MAGeCK

The purpose of MAGeCK, the Model-based Analysis of Genome-wide CRISPR/Cas9 KOs [54], is to prioritize sgRNAs, genes and pathways in genome-scale CRISPR/Cas9 KO screens across different experimental conditions. The various MAGeCK algorithms share a few main steps which start with median normalizing the raw read counts corresponding to sgRNAs. Afterwards, modeling the mean-variance is used to capture the relationship of mean and variance in replicates and a negative binomial (NB) is used to test whether sgRNA abundance differs significantly between treatments (where treatments could be perturbagens or KO time points) and controls (untreated or initial state, T0). This modeling is similar to what has been used for evaluating differential RNA-seq [50–53]. The sgRNAs-level statistics are calculated using the learned NB mean-variance model. The genes targeted by sgRNAs that are ranked consistently higher (by significance) using robust rank aggregation (RRA) [72], are considered the essential genes. The RRA algorithm assumes that if a gene has no effect on selection, then sgRNAs targeting that gene should be uniformly distributed across the ranked list of all the sgRNAs. To rank the genes, RRA compares the skew in rankings according to the uniform null model and prioritizes genes whose sgRNAs’ rankings are consistently higher than expected. Lastly, to identify the enriched pathways the same RRA algorithm is applied to the ranked list of genes. In addition to the original MAGeCK algorithm, MAGeCK-VISPR [56] and MAGeCKFlute [55] are the more recent and updated versions of this algorithm. MAGeCK-VISPR is a workflow that includes a comprehensive quality control (QC), analysis, and visualization workflow for CRISPR screens. This workflow defines a set of QC measures to assess the quality of an experiment and includes a maximum-likelihood algorithm (MLA) (in addition to the original MAGeCK that scores genes based on RRA algorithm) to call essential genes simultaneously under different experimental conditions. The MLA uses a generalized linear model to deconvolute different effects and employs expectation-maximization to iteratively estimate sgRNA KO efficiency and gene essentiality. MAGeCKFlute is an integration of MAGeCK and MAGeCK-VISPR algorithms, with the addition of some QC functions, batch effect removal, copy-number bias correction, and downstream functional enrichment analysis for CRISPR screens. Additionally, it is worth to note that the MAGeCK frameworks include the MAGeCKcount function, which maps the raw FASTQ data to reference library file and count the reads for each sgRNA, which are used for the downstream analysis. Upon its initial development in 2014, MAGeCK has aided in identifying known and novel biologically interesting essential genes and pathways, including EGFR in vemurafenib-treated A375 cells carrying a BRAF mutation, cell-type specific essential genes including BCR and ABL1 in KBM7 cells with a BCR–ABL fusion, and an insulin like growth factor IGF1R in HL-60 cells.

#### STARS

STARS [12] is an algorithm for ranking genes based on their essentiality in genetic perturbation screens. The algorithm takes a list of ranked perturbations (i.e. sgRNAs) as an input. The gene score is computed using the probability mass function of a binomial distribution of the sgRNAs. This is calculated for all sgRNAs that are above the user-defined threshold. The value of the least probable sgRNA for each gene is then assigned to the gene as the STARS score. It is required that at least two sgRNAs rank above the user-defined threshold when scoring a gene to avoid the single sgRNA hits. The null distribution is generated by performing permutation testing on the list of sgRNAs to facilitate the calculation of *P*-values, FDR (false discovery rate) and *q*-values for hit genes. STARS analysis has identified HPRT1 and NUDT5 genes to confer 6-thioguanine resistance in three different cell lines, previously validated genes driving vemurafenib resistance in A375 cells, and modifiers of interferon signaling in mouse BV2 cells.

#### BAGEL

BAGEL [59], or Bayesian Analysis of Gene EssentiaLity, is a supervised learning method for analyzing gene KO screens, trained with gold standard [73] essential and nonessential genes. The initial release of BAGEL (Hart 2016) offered significantly greater sensitivity than the other methods, while BAGEL2 [60] reduced runtime due to the computational optimizations. As an input BAGEL takes the tab-delimited file of sgRNA read counts and reference essential and nonessential gene lists. The first step of BAGEL algorithm is to estimate the distribution of fold changes of sgRNAs targeting all genes in the essential and nonessential training sets. Afterwards, it estimates the likelihood that the observed fold changes for sgRNAs targeting the test gene (withheld from the first step) are drawn from either the essential or nonessential training distributions. The final score for each gene is a log Bayes Factor (BF) which is calculated as the ratio of probabilities that the fold change of the tested gene is drawn from the distribution of essential training set or from the distribution of nonessential training set. The BF score can be calculated for each replicate or at each timepoint if the screen has multiple timepoints. To evaluate the screen performance BAGEL calculates the precision-recall (PR) curves, using the core essential and nonessential gene lists as the test set. The bootstrap resampling of genes in the training set is implemented for validation purposes. BAGEL2 [60] is an improved version of BAGEL, which includes a modeling approach that offers a greater dynamic range of BF, enabling detection of tumor suppressor genes, a multi-target correction that reduces false positives from off-target CRISPR sgRNA, and the implementation of a cross-validation strategy that improves performance ∼10× in comparison with the previous bootstrap sampling approach. In BAGEL2, all genes in the experiment are resampled into training and test sets by 10-fold cross-validation or bootstrapping to calculate the BF. A kernel density estimation is used in each iteration of this sampling to generate the fold change distributions for essential and nonessential genes, using all sgRNAs targeting the control essential or nonessential genes in the resample. The log ratio of essential and nonessential distributions is then taken to generate the log BF on a sgRNA level. A variation of BAGEL, which normalizes gene-level BFs based on the number of gRNA targeting each gene, is used in the Project Score dataset [6].

#### casTLE

Despite the dominance of CRISPR-based screening platform, Morgens et al. developed a Cas9 high-throughput maximum likelihood estimator (casTLE) with the goal of leveraging the data from both RNAi and CRISPR–Cas9 screens. casTLE combines measurements from multiple targeting reagents (e.g. sgRNAs) and the phenotypes of negative controls to estimate the maximum effect size effect for each gene and an associated log-likelihood ratio. In the original study, the casTLE score is presented as twice the log-likelihood ratio. casTLE can be applied on single replicates from different screen types, as well as on combined diverse data types while separately considering experimental noise and variability caused by heterogeneous reagents. The authors of casTLE report that combining the data from RNAi and CRISPR screens led to the improvement in performance in terms of the percent of gold standard essential genes (>85%) at a certain FDR threshold (in this case ∼1% FDR) with an AUC of 0.98 and the identification of ∼4500 nonessential genes. They suggest that this is explained by the RNAi and CRISPR screens revealing different aspects of biology, which is also shown in the low correlation of individual results from the two screens types. To validate casTLE's performance for RNAi and CRISPR screens, casTLE was used to re-analyze data from a few published screens. For each of those screens, casTLE produced results consistent with previous findings, additionally identifying the positive regulators of LPS-induced TNF expression in primary mouse dendritic cells [62].

#### PBNPA

Permutation-based non-parametric analysis (PBNPA) [64] is designed to analyze CRISPR data but can be used to analyze other genetic screens as well. PBNPA computes *P*-values on a gene level by permuting sgRNA labels, thus avoiding restrictive distributional assumptions. To achieve this end-result, PBNPA is designed to accomplish several steps which start with normalizing the raw read counts by multiplying a factor of mean, which makes total read counts in each condition equal. For each sgRNA the natural logarithm gold change is calculated and the median of those is then used as R score for each gene. Gene labels are randomly permuted to form a null distribution of R, which is used to calculate a *P*-value for a gene if it negatively or positively selected gene. After getting the *P*-values for all genes, the small set of genes with *P*-values smaller than a threshold is removed, and the prior permutation process is repeated to get the null distribution with the significant genes removed. Then, the *P*-values are updated for each gene and lastly, Benjamini–Hochberg procedure is used to calculate the FDR values. The authors highlight that using the median log fold change of sgRNAs targeting a gene as the R score for that gene make it more robust against any outliers and influences from potential off-target effects. The authors of PBNPA have used Wang et al. [74] study to validate the performance of their algorithm showing that PNBPA has fewer falsely identifies genes compared with MAGeCK.

### Joint analysis of a panel of screens

#### CERES

CERES is a method to estimate gene dependency from CRISPR KO screens while computationally correcting the copy number effect, therefore enabling unbiased interpretation of gene dependency at all levels of copy number (originally developed based on screens carried out in 342 cancer cell lines) [4]. CERES models the measured sgRNA phenotypic effect or depletion as a sum of gene KO and copy number effects. Additionally, given that the CERES was developed with regards to the 342 cell lines, the total number of cell lines is another determining constant in CERES modeling in addition to the previously three mentioned effects. In CERES, the effect of copy number is modeled by a linear spline, while the gene KO effect is a sum of cell line dependent and independent effects. The sum of these effects is then scaled by a guide activity score, restricted to values between 0 and 1, with the purpose of isolating and mitigating the influence of low-quality sgRNAs. The term accounting for noise, quantification of sgRNA abundance in the reference pool, is also included in the inference model. To infer the mentioned effects and terms, least squares regression is used to model the observed data. CERES gene-level scores are the inferred gene KO effects which are scaled per cell line such that scores of 0 and −1 represent the median effects or nonessential and core essential genes, respectively.

#### JACKS

The Joint Analysis of CRISPR Knockout Screens (JACKS) is a Bayesian method that analyzes panels of screens performed with the same guide RNA library [67]. It is designed to address the signal variability from different sgRNAs that target the same gene, which confounds gene effect estimation and dictates large experiment sizes. JACKS defines the observed log_2_ fold change of an sgRNA as the mean across median-normalized replicate measurements, which is modeled as a Gaussian distribution based on condition-dependent gene effect, condition-independent sgRNA efficacy and the precision parameter of log_2_ fold change which uses a non-parametric approach to assign an empirical Bayes prior that accounts for mean-dependent variability of the log_2_ fold change within the replicate measurements. To overcome the poor direct estimates of these variances in usual cases where the CRISPR screens are carried out with 2–3 replicates, JACKS computes a smoothed mean-dependent estimate of this empirical variance based on all sgRNAs in each condition and then assigns the priors on the precision parameter. To infer the posterior distributions of the three mentioned parameters (condition-dependent gene effect, condition-independent guide effect, and precision) variational inference is used. *P*-values on gene-level are derived from a non-parametric distribution of condition-independent guide effects computed by running JACKS inference on a number of sgRNAs (from the negative controls set, i.e. guide targeting nonessential genes or cutting only in noncoding regions) targeting randomly selected test genes.

## Conclusions and future directions

As illustrated above, a variety of computational tools are available for analysis of CRISPR screens. These tools use a variety of approaches to identify genes undergoing negative selection in a pooled library screen. Only a subset, however, take into account the quality of the underlying data. The reagents and enzymes behind CRISPR screens have evolved tremendously over the last half-decade, and high signal to noise is now the expectation for a modern CRISPR library in a tractable model. With high data quality, choice of analytical approach is less important, as most approaches will give highly concordant results and marginal hits are likely less interesting because of their lower KO fitness defects. Quality control remains a critical first step.

Copy number effect influences the measurement of the gene deletion effect on cell survival and it can be detected even among low-level copy number amplifications and deletions [5]. Therefore, correcting for copy number [75] is an important step in processing and analyzing CRISPR screens. CRISPRcleanR [70] and CRISPy [71] are two single sample-based methods for correcting the copy number effect, contrary to CERES which performs correction across multiple samples. CRISPRclearR corrects the gene-independent responses in genome-wide CRISPR KO screens in an unsupervised manner, whereas CRISPy corrects it in a supervised way using segment level copy number ratios. CRISPRcleanR is library agnostic, and as results outputs sgRNA fold changes and normalized read counts, which makes it compatible with downstream analysis tools, such as BAGEL or MAGeCK, for hit calling and interpretation of gene KO phenotypic effects.

Going forward, CRISPR editing will continue to be a fast-moving field, with new perturbation modalities demanding development of new analytical approaches. In this context, it is worth highlighting the presence of Cas12a-based screening platforms. The Cas12a nuclease, which can process multiple gRNA from a single polycistronic transcript, offers an attractive alternative to Cas9 for multiplex screening, which facilitates the large-scale investigation of genetic interactions in mammalian cells (Figure 1F). In the DeWeirdt et al. study, which optimized libraries for the engineered enCas12a variant, the group also screened for synthetic lethality in two cancer cell lines (OVCAR8 and A375) and discovered previously unreported interaction between MARCH5 and WSB2 [76]. Dede et al. has utilized the enCas12a platform to investigate the functional buffering among ∼400 candidate paralog pairs in three cell lines. The authors observed 24 synthetic lethal paralog pairs that were previously undetected by monogenic KO screens. The Moffat group took advantage of the Cas12a system in a different fashion — combing Cas9 and Cas12a to create a hybrid Cas platform, CHyMErA, to evaluate a set of 672 human paralog pairs, and explore chemogenetic interactions in the mTOR pathway [77]. These and other similar studies indicate the need for robust methods specifically designed to analyze and quantify the mutual phenotypic effects of simultaneous digenic or higher-order KOs. There are several computational methods for scoring digenic KOs CRISPR screens [40,41,78–81], however only two of them [80,81] are generalized and available as R packages.

## Summary

- The advent of CRISPR technology has revolutionized the cancer biology by providing insights about cancer treatment strategies through the discovery of essential genes for drug targets, identification of metastatic regulators, drug resistance mechanisms, immunotherapy targets, and synthetic lethality.
- There exists a number of bioinformatics tools and methods for the analysis of CRISPR screens based on different mathematical models and being continuously optimized.
- As CRISPR versatility expands and technologies continue to develop, we foresee the need for the development of methods specifically designed to robustly quantify these novelties and provide the functional characterizations for genetic systems of interest.

## Abbreviations

BF: Bayes Factor
FDR: false discovery rate
JACKS: Joint Analysis of CRISPR Knockout Screens
KO: knockout
MLA: maximum-likelihood algorithm
NB: negative binomial
PBNPA: Permutation-based non-parametric analysis
QC: quality control
RRA: robust rank aggregation
